# Cofactor engineering to regulate NAD^+^/NADH ratio with its application to phytosterols biotransformation

**DOI:** 10.1186/s12934-017-0796-4

**Published:** 2017-10-30

**Authors:** Liqiu Su, Yanbing Shen, Wenkai Zhang, Tian Gao, Zhihua Shang, Min Wang

**Affiliations:** Key Laboratory of Industrial Fermentation Microbiology, Ministry of Education, College of Biotechnology, Tianjin University of Science & Technology, Tianjin, 300457 People’s Republic of China

**Keywords:** Phytosterols biotransformation, Proteomic analysis, NADH: flavin oxidoreductase, NADH oxidase, *Mycobacterium neoaurum*

## Abstract

**Background:**

Cofactor engineering is involved in the modification of enzymes related to nicotinamide adenine dinucleotides (NADH and NAD^+^) metabolism, which results in a significantly altered spectrum of metabolic products. Cofactor engineering plays an important role in metabolic engineering but is rarely reported in the sterols biotransformation process owing to its use of multi-catabolic enzymes, which promote multiple consecutive reactions. Androst-4-ene-3, 17-dione (AD) and androst-1, 4-diene-3, 17-dione (ADD) are important steroid medicine intermediates that are obtained via the nucleus oxidation and the side chain degradation of phytosterols by *Mycobacterium*. Given that the biotransformation from phytosterols to AD (D) is supposed to be a NAD^+^-dependent process, this work utilized cofactor engineering in *Mycobacterium neoaurum* and investigated the effect on cofactor and phytosterols metabolism.

**Results:**

Through the addition of the coenzyme precursor of nicotinic acid in the phytosterols fermentation system, the intracellular NAD^+^/NADH ratio and the AD (D) production of *M. neoaurum* TCCC 11978 (*MNR* M3) were higher than in the control. Moreover, the NADH: flavin oxidoreductase was identified and was supposed to exert a positive effect on cofactor regulation and phytosterols metabolism pathways via comparative proteomic profiling of *MNR* cultured with and without phytosterols. In addition, the NADH: flavin oxidoreductase and a water-forming NADH oxidase from *Lactobacillus brevis*, were successfully overexpressed and heterologously expressed in *MNR* M3 to improve the intracellular ratio of NAD^+^/NADH. After 96 h of cultivation, the expression of these two enzymes in *MNR* M3 resulted in the decrease in intracellular NADH level (by 51 and 67%, respectively) and the increase in NAD^+^/NADH ratio (by 113 and 192%, respectively). Phytosterols bioconversion revealed that the conversion ratio of engineered stains was ultimately improved by 58 and 147%, respectively. The highest AD (D) conversion ratio by *MNR* M3N2 was 94% in the conversion system with soybean oil as reaction media to promote the solubility of phytosterols.

**Conclusions:**

The ratio of NAD^+^/NADH is an important factor for the transformation of phytosterols. Expression of NADH: flavin oxidoreductase and water-forming NADH oxidase in *MNR* improved AD (D) production. Besides the manipulation of key enzyme activities, which included in phytosterols degradation pathways, maintenance the balance of redox also played an important role in promoting steroid biotransformation. The recombinant *MNR* strain may be useful in industrial production.

## Background

Metabolic engineering studies generally focused on genetic manipulation (overexpression or disruption) of the genes that encode enzymes involved in a particular pathway. However, the flux of a cofactor-dependent pathway is controlled through the availability of enzyme, as well as the cofactor and ratio of the reduced to the oxidized form of the cofactor. Therefore, cofactor manipulation is a potentially powerful tool for metabolic engineering [1]. Cofactor engineering, a subset of metabolic engineering, is defined as the manipulation of the cofactors in metabolic pathways and optimize dynamic control of the target metabolic flux. It has been successfully applied in *Escherichia coli* (*E. coli*) [2, 3], *Lactococcus lactis* [1], *Saccharomyces cerevisiae* [4], *Klebsiella pneumoniae* [5], *Serratia marcescens* [6], *Torulopsis glabrata* [7], and *Colletotrichum lini* [8]. Nicotinamide adenine dinucleotides (NADH and NAD^+^) is an important cofactor pair that acts in plenty of oxidation–reduction (redox) reactions and regulates various enzyme activities and genetic processes [5–7, 9]. Therefore, the cofactor pair NAD^+^ and NADH has a critical effect on the maintenance of the intracellular redox balance, which is a basic condition for the growth and metabolism of microorganisms [4]. The NAD^+^ manipulation system has been successfully used to improve the production of primary metabolites such as ethanol, 1, 2-propanediol [10], acetoin [5, 6, 11], and pyruvate [7]. The most effective way of adjusting the NAD^+^/NADH ratio is by introducing an NADH or NAD^+^ regeneration system. Intracellular NADH or NAD^+^ is easily regenerated in situ by expressing an NAD^+^-dependent formate dehydrogenase (increase of intracellular NADH availability) or an NADH oxidase (high NAD^+^/NADH ratio), respectively [6, 12]. Furthermore, the overexpression of the gene of *pncB*, which encodes NAPRTase, results in increased total NAD^+^ level and ratio of NAD^+^/NADH [2].

Steroid medications are used widely in clinical applications and form an important and large category in the pharmaceutical industry. Androst-4-ene-3, 17-dione (AD) and androst-1, 4-diene-3, 17-dione (ADD) are two versatile C19 steroid precursors that are obtained via the nucleus oxidation and the side chain degradation of phytosterols by microorganisms [13, 14]. The process of AD (D) production was carried out using multi-catabolic enzymes promoting many consecutive reactions and not all enzymes included in this complicated process were well studied so far. In general, the manipulation of the key enzyme activities included in phytosterols nucleus oxidation or the side chain cleavage pathways could reportedly enhance the AD (D) production, such as the deletion of 3-ketosteroid-1-dehydrogenase [15, 16], or the overexpression of cholesterol oxidase [17], 3β-hydroxysteroid dehydrogenases [18] and 3-ketosteroid-9α-hydroxylase [15]. However, the overexpression, deletion, or introduction of heterologous genes in target metabolic pathways does not always result in the desired phenotype [19]. Given the multi-catabolic enzyme biotransformation of phytosterols into AD, the cofactors are closely related to phytosterols side-chain degradation. As postulated, the side chain with one mole of sitosterol is selectively removed, as follows: β-sitosterol + 21 H_2_O + 4 ATP + 7 GDP + 7 P_i_ + 10 FAD + 21 NAD^+^ = AD (D) + 21/2 CO_2_ + 4 AMP + 4 PP_i_ + 7 GTP + 10 FADH_2_ + 21 NADH + 21 H^+^ [20, 21]. Maintenance of the redox balance or the regeneration activity of cofactors is supposedly a rate-limiting factor in steroid synthesis. However, given the complication of phytosterols biotransformation pathways, studies on the relationship of cofactors or NAD^+^/NADH ratio to the AD (D) production are few.

In this work, cofactor manipulation was used in phytosterols biotransformation. First, the connection of phytosterols biotransformation with the intracellular NAD^+^/NADH ratio in *Mycobacterium neoaurum* TCCC 11978 (*MNR* M3) was analyzed by adding nicotinic acid (NA) in the broth. Then, the key enzymes that related to NAD (H) regulation and phytosterols degradation were identified according to the analysis of comparative proteomic of *M. neoaurum* cultured with and without phytosterols. Afterward, the cofactor engineering was conducted by introduction of NAD^+^ regeneration system into *MNR* M3 to increase the availability of NAD^+^/NADH ratio for AD (D) production based on the proteomic analysis.

## Methods

### Strain and plasmid construction

The strains, plasmids, and primers used in this study are listed in Table 1.

**Table 1:** Bacterial strains, plasmids and primers used in this study

Strains, plasmids, and primers	Significant properties	Source or purpose
Strains		
*Mycobacterium neoaurum* TCCC 11028 M3 (*MNR* M3)	Wild type	Tianjin University of Science and Technology Culture Collection Center (TCCC)
*Lactococcus lactis* subsp. cremoris NZ9000	Source of the *nox* gene	Tianjin University of Science and Technology Culture Collection Center (TCCC)
*E. coli* DH5a	General cloning host	Transgen Biotech
*MNR* M3N1	*MNR* M3 containing plasmid pMV261-*nox*-1	This work
*MNR* M3N2	*MNR* M3 containing plasmid pMV261-*nox*-2	This work
Plasmids		
pMV261	*Mycobacterial* replicating vector carrying the BCG hsp60 promoter, kan	Dr. W. R. Jacobs Jr. (Howard Hughes Medical Institute), for providing plasmid pMV261
pMV261-*nox*-1	pMV261, contain *nox* gene from *MNR* M3, hsp60, kan, *Bam*HI/*Hin*dIII	This work
pMV261-*nox*-2	pMV261, contain *nox* gene from *Lactococcus lactis* subsp. cremoris NZ9000, hsp60, kan, *Bam*HI/*sal*I	This work
Primers		
16 s rRNA-f-RT	ACCAGCGTCCTGTGCATGTC	Quantitative RT-PCR
16 s rRNA-r-RT	AGTACGGCCGCAAGGCTAAAAC	Quantitative RT-PCR
Nox-1-f-RT	GGAACAGGTACATGGGGTTG	Quantitative RT-PCR for *nox*-1
Nox-1-r-RT	GAAGTGGCTGGAAGAAGACG	Quantitative RT-PCR for *nox*-1
*nox*-1-f	CGCGGATCCAATGAACACCCAGCCGAAAGT	*nox*-1 amplification
*nox*-1-r	CCCAAGCTTTCAGACCGTGAGGGTGTCCG	*nox*-1 amplification
*nox*-2-f	5′-CGGGGATCCGAAAATCGTAGTTATCGGTA-3′	*nox*-2 amplification
*nox*-2-r	5′-GCGTCGACTTATTTGGCATTCAAAGCTG-3′	*nox*-2 amplification
PMV-f	5-TAGGCGAGTGCTAAGAATAACGTTG-3	Amplification

*Mycobacterium neoaurum* TCCC 11978 (*MNR* M3) obtained from Tianjin University of Science and Technology Culture Collection Center (TCCC), Tianjin, China, was a spontaneous mutant of *M. neoaurum* TCCC 11028 (*MNR*) strain. AD accumulated in the broth as a major product by *MNR* M3. The amount of ADD was too low, less than 5% of the total product in the medium. The AD (D) concentration is the total sum of the two products. *MNR* M3C2 is the *ksdD* gene replacement strain *MNR* M3*ΔksdD::ksdD*-*MNR* which was constructed by homologous recombination [16]. The *nox*-1 gene was amplified through the polymerase chain reaction from the total DNA of *MNR* M3, using the primers *nox*-1-f and *nox*-1-r that generate *Bam*HΙ and *Hin*dIIΙ sites. The *nox*-2 gene was amplified from the total DNA, which was isolated from *Lactococcus lactis* subsp. cremoris NZ9000 (*L. lactis* subsp. cremoris NZ9000), using the primers *nox*-2-f and *nox*-2-r that generate *Bam*HΙ and *Sal*Ι sites. Mycobacterial replicating vector pMV261 which harbored the Kanamycin (*kan*) resistance was the common plasmids used in *Mycobacterium* for gene expression [22]. The amplified fragments were digested by *Bam*HI and *Hin*dIII or *Bam*HI and *Sal*I, respectively. Then the treated fragments were inserted into the vector pMV261, which was treated with the same restriction enzymes to generate the vector pMV261-*nox*-1 and pMV261-*nox*-2. The pMV261-*nox*-1 and pMV261-*nox*-2 vectors were imported into *MNR* M3 via electroporation. The transformants were screened in a Luria–Bertani medium agar plate containing *kan*. The selected recombinants were then designated as *MNR* M3N1 and *MNR* M3N2 for further characterization.

### Chemicals and culture conditions

The phytosterols substrate used is a sterol mixture contained (by weight percentage) 51.7% β-sitosterol, 27.2% stigmasterol, 17.1% campesterol, and 4.0% brassicasterol (COFCO Tech Bioengineering Co., Ltd., Tianjin). Standards AD and ADD were purchased from Sigma-Aldrich Co. (USA). All chemical solvents and salts were of analytical grade or higher. The cultivation and bioconversion of microorganisms and the preparation and analysis of transformation products, were performed following the procedures described by Shen et al. [23]. The minimal medium contained (g/L): glucose 10, MgSO_4_ 0.5, K_2_HPO_4_ 0.5, (NH_4_)_2_HPO_4_ 3.5, citric acid 2, and ammonium iron citrate 0.05 with pH of 7.2. Experiments were conducted under different culture conditions with phytosterols (5 g/L) or soybean oil (16%) and Tween 80 (0.5%) in minimal medium. Different concentrations of NA were added to the medium at the start of fermentation. In the phytosterols-free medium, the growth of cell was measured through optical density. However, the cell growth in the phytosterols-contained culture broth is difficult to measure by using this method. In this study, the cell growth measurement in the medium with phytosterols was conducted following the method by Meyers et al. [24]. The amount of protein was related to the dry cell weight (DCW) obtained using an adequate calibration curve: ${\text{DCW }}\left( {{\text{mg}}/{\text{mL}}} \right) = 2.10 \times {\text{ protein }}\left( {{\text{mg}}/{\text{mL}}} \right) - 0.17 \left( {{\text{R}}^{2} = 0.99} \right).$ The growth of cell was represented by DCW. All experiments were performed in triplicate and the data were statistically analyzed by one-way ANOVA.

### Determination of NAD^+^ and NADH concentrations

The intracellular concentrations of NADH and NAD^+^ were determined as described by Zhang et al. [12].

### Assay of NOX activity

0.2 g wet cells of the strains were washed with Tris–HCl buffer (50 mM Tris–HCl, pH 7.5, 0.2 M NaCl, 1% Triton X-100) twice, and resuspended in the same buffer. Cells were then disrupted by sonication at 4 °C for 10 min by a cell sonicator. The homogenate was centrifuged at 12,000 r/min for 30 min at 4 °C. The supernatant was stored in − 80 °C for further research. NOX activity was determined through a photometer assay at 340 nm using 0.1 mM NADH and 0.1 M potassium phosphate buffer at pH 7.0. The reaction was initiated by adding 0.1 mL of cell extracts to the 0.9 mL reaction mixture, and the decrease in absorbance at 340 nm was determined to calculate the NADH concentration. A unit of NOX activity is defined as the amount that catalyzes the oxidation of 1 μmol NADH to NAD^+^ per minute. Protein concentrations were measured by the method described by Bradford.

### Protein analysis and identification

*MNR* M3C2 was grown in minimal medium. Experiments were conducted under different culture conditions with phytosterols and without phytosterols in minimal medium. The cells were harvested after 60 h cultivation. The collected sediment was washed with phosphate buffer saline at pH 7.2 thrice, sonicated on ice using a high intensity ultrasonic processor (Scientz) in lysis buffer (8 M Urea, 50 mM Tris -HCl, pH 7.5, 1% Nonidet P 40, 1% Sodium deoxycholate, 2 mM Ethylenediaminetetraacetic acid, 5 mM Dithiothreitol, 1% Protease inhibitor), and centrifuged at 20,000*g* for 10 min at 4 °C. The protein contents in supernatural fluids were estimated using 2D Quant kit. Proteins were prepared and analyzed using tandem mass tag (TMT)—based LC–MS/MS. More specifically, the protein solution was reduced with 5 mM dithiothreitol for 1 h at 37 °C and alkylated with 20 mM iodoacetamide for 45 min at room temperature in darkness. For trypsin digestion, the protein sample was diluted by adding 100 mM triethylamine borane. Finally, trypsin was added at 1:50 trypsin-to-protein mass ratio for the first digestion overnight and 1:100 trypsin-to-protein mass ratio for a second 4 h-digestion. Approximately 300 μg protein for each sample was digested with trypsin for the following experiments. After trypsin digestion, peptide was desalted by Strata X C18 SPE column (Phenomenex) and vacuum-dried. Peptide was reconstituted in 0.5 M triethylamine borane and processed according to the manufacturer’s protocol for 6-plex TMT kit (Pierce). TMT-labeled samples were diluted to 10 mM HCOONH_4_ buffer (NH_3_H_2_O, pH 10) before HPLC on Waters XBridge Shield C18 RP column, 3.5 μm, 4.6 × 250 mm. The flow rate used for reversed-phase column separation is 1 mL/min with mobile phase A (10 mM HCOONH_4_) and mobile phase B (10 mM HCOONH_4_, 80% ACN). A solvent gradient system was used: 0–5 min, 2–10% B; 5–55 min, 10–35% B; 55–65 min, 35–90% B; 65–70 min, 90% B; 70–75 min, 90–2% B; 75–80 min, 2% B. In total, 20 fractions were pooled for each sample and dried by vacuum centrifuge. The peptides were separated by a linear gradient formed from 2% ACN, 0.1% FA (mobile phase A), and 80% ACN, 0.1% FA (mobile phase B). A solvent gradient system was used: 0–5 min, 2% B; 5–8 min, 2–11% B; 8–53 min, 11–20% B; 53–55 min, 20–80% B; 55–58 min, 80% B; 58–60 min, 80–2% B; 60–65 min, 2% B. at a flow rate of 300 μl/min. MS analysis was performed on a Thermo Scientific Q Exactive plus. The electrospray voltage applied was 2.0 kV. MS spectra were acquired across the mass range of 350–1500 m/z in high resolution mode using 250 ms accumulation time per spectrum. For data analysis, raw data (.raw) was converted into peak lists (.mgf) by Proteome Discoverer. The database used in searching was uniprot reference proteome *M. neoaurum* VKM Ac-1815D (Uniprot: UP000018763) [25]. Mass error was set to 20 ppm for precursor ions and 0.02 Da for fragment ions. Carbamidomethylation on Cys was specified as fixed modification and oxidation on methionine was specified as variable modifications. Protein quantification data with relative expressions of > 1.5 and < 0.67 and *p* values of < 0.01 were selected to ensure the authenticity of up- and down-regulations. Gene ontology (GO) (http://www.geneontology.org/) was used to investigate the potential functions of peptide precursors. The ontology covers three domains, as follows: cellular component, molecular function, and biological process. Kyoto Encyclopedia of Genes and Genomes (KEGG) database (http://www.genome.jp/kegg/) was used to annotate the protein pathway.

### Isolation of RNA and quantitative RT-PCR analyses

*MNR* M3 was grown under different culture conditions with phytosterols and without phytosterols in minimal medium. The cells were harvested after 60 and 72 h cultivation (late logarithmic phase) and preserved at − 80 °C. Total RNA was isolated using Eastep® Super Total RNA Extraction Kit (Promega, Shanghai) according to instructions of supplier. The resulting mixture was treated with DNAse I. For reverse transcription, cDNA synthesis was performed using the PrimeScript™ RT reagent Kit with gDNA Eraser (Perfect Real Time) (Takara) with 0.3 μg of total RNA following the manufacturer’s instructions. Quantitative RT-PCR analyses of cDNA samples were performed on the StepOneTM RealTime PCR System (Applied Biosystems). The above cDNA sample was completely mixed with 0.2 μM specific oligonucleotides, 10 μL of AceQ® qPCR SYBR® Green Master Mix, and 0.4 μL of ROX Reference Dye 1 in 20 μL of reaction mixture. The nucleotide sequences of primers used in this study for target and reference genes are listed in Table 1. Quantitative RT-PCR analyses were performed as follows: 5 min pre-denaturing, 40 cycles of 95 °C for 10 s, 60 °C for 30 s, followed by melting curve stage from 60 to 95 °C. Each gene was measured in triplicate from three independent tests. The cDNA amplification efficiency of samples, internal standards (16S rRNA), and calibrators (samples without induction of phytosterols) were equivalently modulated to ensure that the relative amount of mRNA could be processed using the 2^−ΔΔCt^ algorithm [26]. Each gene was measured in triplicate from three independent tests.

## Results

### Interaction between phytosterols degradation process and the intracellular NAD^+^/NADH ratio

To better understand the connection of the intracellular NAD (H) with AD (D) production in *MNR* M3, the intracellular NAD^+^/NADH ratio and AD (D) production during biotransformation were examined at regular intervals; on the other hand, NA (vitamin precursor of NAD^+^) was added to the fermentation system to change the intracellular redox status.

**Fig. 1:**
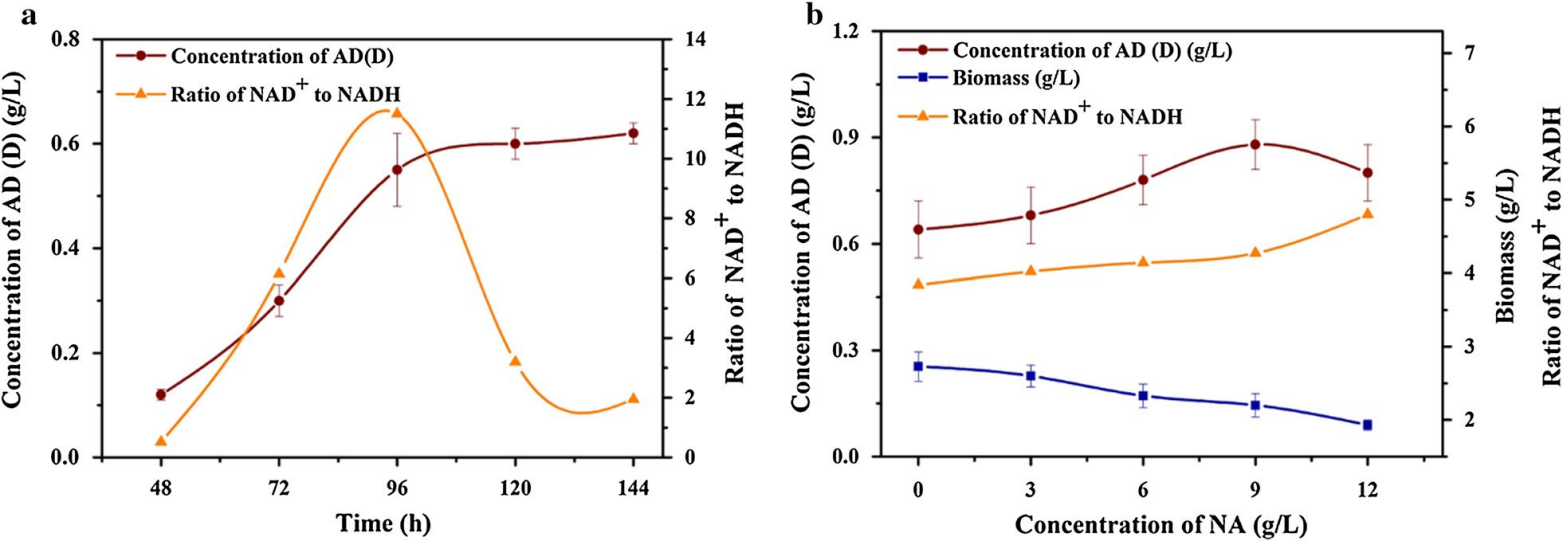
**a** Time courses of the AD (D) production and NAD^+^/NADH ratio during phytosterols transformation with *MNR* M3. **b** Effect of NA on cell growth, NAD^+^/NADH ratio, and AD (D) production during phytosterols biotransformation with *MNR* M3. Biotransformation conditions: 50 mL fermentation medium in 250 mL shake flask, 5 g/L phytosterols, 30 °C, 140 r/min for 120 h transformation. The *error bars* represent mean ± SD (n = 3)

As shown in Fig. 1a, the ratio of NAD^+^/NADH constantly changed during the fermentation. This outcome is consistent with the studies of Ji et al. [5] and Wu et al. [8]. During the stage from 48 to 96 h, the NAD^+^/NADH ratio in *MNR* M3 increased. Following, the NAD^+^/NADH ratio decreased quickly after 96 h and the highest NAD^+^/NADH ratio was obtained at 96 h. The dynamics of the accumulation of AD (D) was also studied. From 48 to 72 h, the productivity of AD (D) is 7.5 mg/(L h), which increased to 10.4 mg/(L h) at the next stage (72–96 h). After 96 h, the rate remarkably decreased to 2.9 mg/(L h). The productivity of AD (D) showed a similar trend with the change of NAD^+^/NADH ratio. Thus, we preliminarily inferred a correlation between the NAD^+^/NADH ratio and the AD (D) productivity during the late biotransformation period (after 96 h) in *MNR* M3.

NA is used to elevate the intracellular NAD^+^ level through the salvage pathway in *M. tuberculosis* [27]. A suitable concentration of NA in the medium is important for the efficient production [2, 28]. Thus, a series of NA concentrations with phytosterols was simultaneously added to the fermentation medium of *MNR* M3. From Fig. 1b, the AD (D) production was continuously increased with increasing NA concentration (0–9 g/L), and the highest level of AD (D) (0.88 g/L) was achieved with 9 g/L NA. This value was 37.5% higher than in the control which without NA (0.64 g/L). The addition of NA could increase the NAD^+^/NADH ratio during the late biotransformation period as well. When 12 g/L NA was added into the fermentation system, the level of NAD^+^/NADH ratio was 25% higher than that of the control (without NA). However, the weak growth was observed with increasing NA concentration in the fermentation medium, which is in accordance with that in *Colletotrichum lini* [8]. The significantly inhibition of the cell growth at high concentration of NA might lead to the AD (D) production with 12 g/L NA was lower than that with 9 g/L NA. As stated above, high levels of NAD^+^/NADH ratio was obtained and the production of AD (D) was enhanced with the addition of NA. We could get that the regeneration activity of NAD^+^ had a positive effect on the production of AD (D).

### Selection of the gene targets for NAD^+^ and NADH modification to enhance the AD (D) production

It’s necessary to establish an efficient way to generate NAD^+^ cofactor by genetic modification in *MNR* M3. In order to investigate whether the proteins regulate the redox balance of cofactor and bring a positive influence on phytosterols metabolic pathway, we detected the changes in proteome of *MNR* M3C2 cultured with and without phytosterols in minimal medium.

A total of 19025 peptides and 3727 proteins were identified, among which 299 proteins showed significant changes, as follows: 174 proteins were up-regulated and 125 proteins were down-regulated. The changed proteins included in cofactor metabolism were analyzed. The accession number, protein name, and fold change (with phytosterols/without phytosterols) for proteins were available in Table 2. The regeneration of NAD^+^ from NADH was mainly through electron transfer chain (ETC), oxygen was used as the final electron acceptor and ATP was produced [7]. So the proteins involved in ETC were analyzed, the NADH dehydrogenase (NuoL, NuoG, NuoC, and NuoE) were down-regulated obviously due to the existence of phytosterols. This finding is consistent with the study stating that sterol substrates and products such as ADD and AD inhibit the cell growth and impair the cell’s respiratory chain [29, 30]. Apart from the ETC pathway, expression of an NADH: flavin oxidoreductase (NOX-1), which can oxidize NADH using O_2_ to produce NAD^+^ and H_2_O, was also detected in *MNR* M3C2. It was noteworthy that the expression of this protein was significantly up-regulated by 3.1-fold with phytosterols in minimal medium. By analyzing the proteome of *MNR* M3 with and without phytosterols, we speculated that the up-regulated protein NOX-1 performed important functions in phytosterols metabolism and cofactor regulation pathways.

**Table 2:** Identified differentially expressed proteins of *MNR* M3C2 in the presence of phytosterols

Protein accession	Protein description	Changed ratio
V5X8K9	NADH-quinone oxidoreductase subunit L	0.497
V5X8L5	NADH-quinone oxidoreductase subunit G	0.561
V5XBK6	NADH-quinone oxidoreductase subunit C	0.501
V5X9W2	NADH-quinone oxidoreductase subunit E	0.479
V5X9K2	NADH:flavin Oxidoreductase	3.115

Changed ratio presents the ratio of proteins with phytosterols in the fermentation medium to that without phytosterols

**Fig. 2:**
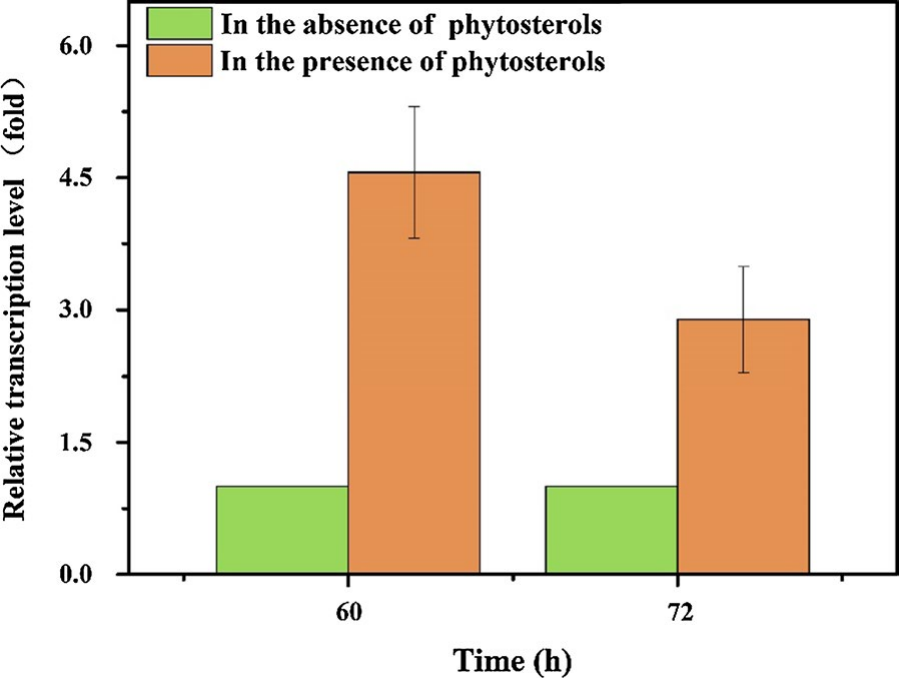
Transcription levels of NADH: flavin Oxidoreductase in *MNR* M3 induced by phytosterols

To validate the results from proteomic analysis, the transcription level of NOX-1 in *MNR* M3 with and without phytosterols was assayed by quantitative RT-PCR. As shown in Fig. 2, when compared with that of *MNR* M3 cultured without phytosterols, the relative transcription of NOX-1 increased by 4.56 and 2.89 times after cultivation for 60 and 72 h, respectively. This finding indicated the credibility of the up-regulated ratio of NOX-1 with phytosterols showed in proteomic analysis.

### Expression of NOX in *MNR* M3 and its effect on NAD^+^/NADH ratio

Both NOX-1 and NADH oxidase (NOX-2) catalyze the oxidation of NADH to NAD^+^ by using molecular oxygen as the electron acceptor [31, 32]. In order to improve the NAD^+^/NADH ratio for phytosterols biotransformation, NOX-1 was overexpressed in *MNR* M3. Moreover, the NOX-2 from *L. lactis* subsp. cremoris NZ9000 was also chosen to express in *MNR* M3 as it can lead to a dramatically increased NAD^+^/NADH ratio [33, 34]. As stated in the Materials and Methods Section, the confirmed recombinants *MNR* M3N1 and *MNR* M3N2 were obtained.

The specific activity of NOX and the content of NAD^+^ and NADH were measured when the cells entered the end stage of exponential phase. As shown in Table 3, the specific activity of NADH oxidase (0.86 U/mg protein) in the recombinant strain *MNR* M3N2 was higher than that of *MNR* M3N1 (0.32 U/mg protein), whereas the NADH oxidase activity in the parent strain was not detected. This finding indicated that NOX was actively produced in the recombinant strains, and the expression of NOX-2 in *MNR* M3N2 had a higher catalytic activity.

**Table 3:** Intracellular NADH oxidase activities, NAD (H) concentrations and ratio of NAD^+^ to NADH in *MNR* M3, *MNR* M3N1 and *MNR* M3N2

Strains	*MNR* M3	*MNR* M3N1	*MNR* M3N2
NADH oxidase activity (U/mg)	ND	0.32 ± 0.02	0.86 ± 0.02
NAD^+^ (μmol/g DCW)	9.34 ± 0.50	9.71 ± 0.33	9.11 ± 0.72
NADH (μmol/g DCW)	0.90 ± 0.15	0.44 ± 0.05	0.30 ± 0.02
Ratio of NAD^+^ to NADH	10.4	22.1	30.4

Biotransformation conditions: 50 mL fermentation medium in 250 mL shake flask, 30 °C, 140 r/min, 96 h. All experiments were performed in triplicate

**Fig. 3:**
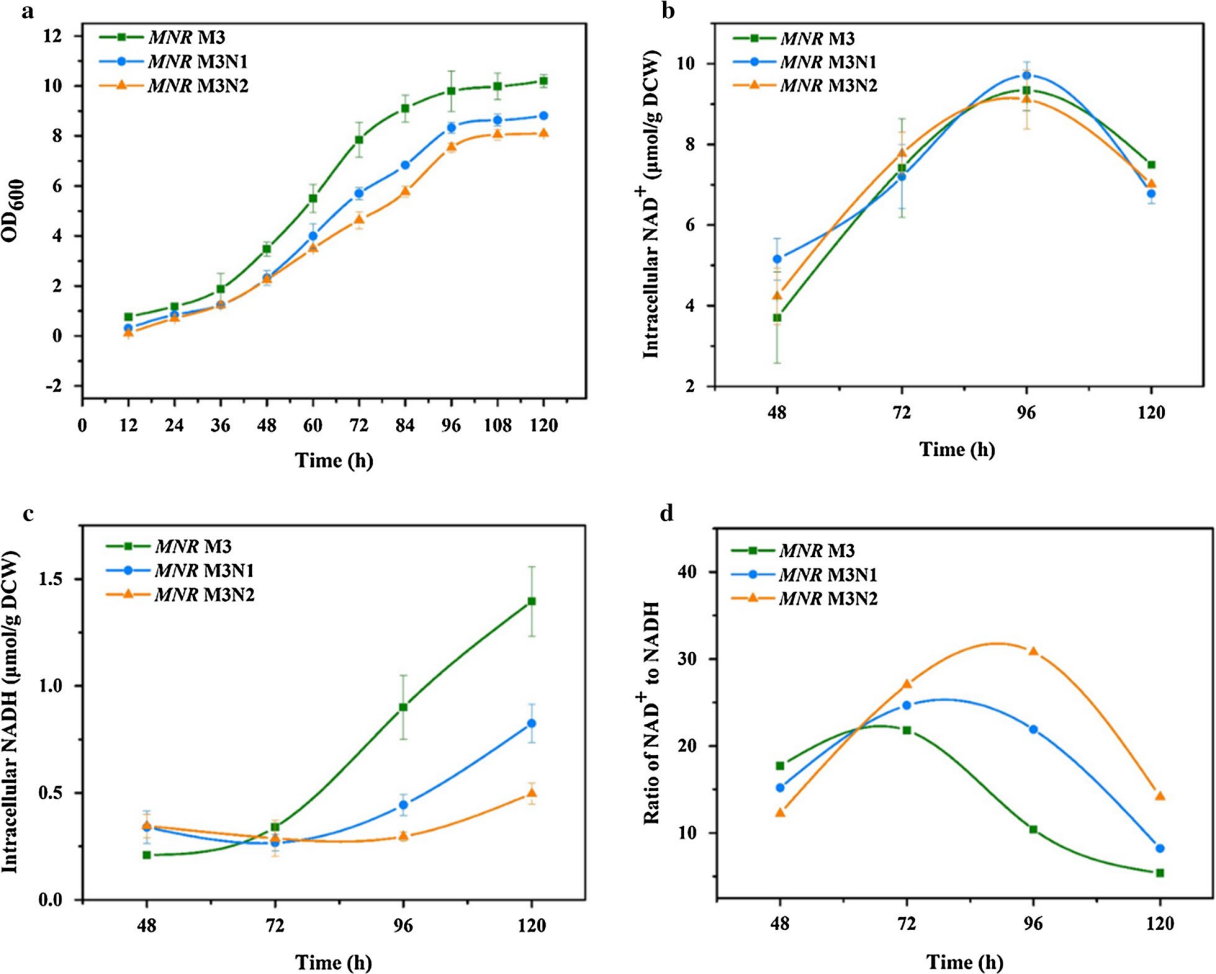
Time courses of various parameters during cultured in minimal medium with *MNR* M3, *MNR* M3N1*, MNR* M3N2, respectively. It shows the cell growth (**a**), the concentrations of intracellular NADH (**b**), NAD^+^ (**c**), and NAD^+^/NADH ratio (**d**). Biotransformation conditions: 50 mL fermentation medium in 250 mL shake flask, 30 °C, 140 r/min for 120 h culture. The *error bars* represent mean ± SD (n = 3)

The effect of NOX expression on cell growth was studied during aerobic cultivations of strains *MNR* M3, *MNR* M3N1, and *MNR* M3N2. As shown in Fig. 3a, cell growth trends of these strains shared typical curves that could be divided into four stages, as follows. They grew slowly at the first 36 h. Then, they grew fast (36–84 h). The growth rates were lowered down (84–96 h) and cell concentrations were unchanged after 96 h of cultivations. However, the growth rate of the NOX expression strains were slower than that of the parent strain, the growth rate of *MNR* M3N1 was better than that of *MNR* M3N2 with higher NOX expression. The NOX expression had a negative effect on cell growth.

The expression of NOX in *MNR* M3 was expected to increase the overall intracellular NAD^+^ pool and NAD^+^/NADH ratio, thereby improving the flux of NAD^+^-dependent pathways. As shown in Fig. 3b, c, intracellular concentrations of NADH and NAD^+^ continuously changed during various stages of the cell growth process among the parent and the engineered strains. In the three strains, levels of NAD^+^ were increased during growth phases and when cells entered non-growth phase, the NAD^+^ levels in these three strains were decreased. No remarkable difference on the NAD^+^ level among these three strains was observed. However, levels of NADH in the three strains were different. The NADH levels remain unchanged at the first 72 h, and when cells entered the end stage of exponential and the non-growth phase, the NADH levels in these three strains were increased. The increased rate of NADH level in the engineered strains was lower than that in the parent strain, and *MNR* M3N2 with the higher NOX expression has the lowest increase of NADH level among these three strains. NOX expression led to a decrease of NADH pools comparing with the parent strain. Meanwhile, the ratio of NAD^+^/NADH ratio among these three strains was studied (Fig. 3d). The production of NOX in *MNR* M3 led to the increase of NAD^+^/NADH ratio. After 96 h, the ratio in *MNR* M3N1 and *MNR* M3N2 was 113 and 192% higher than that in the parent strain, respectively. This suggests that the redox balance in *MNR* M3 producing NOX was disturbed, which also explains why cell growth of *MNR* M3N1 and *MNR* M3N2 was affected (Fig. 3a).

### Effect of NOX expression on AD (D) production in the fermentation

**Fig. 4:**
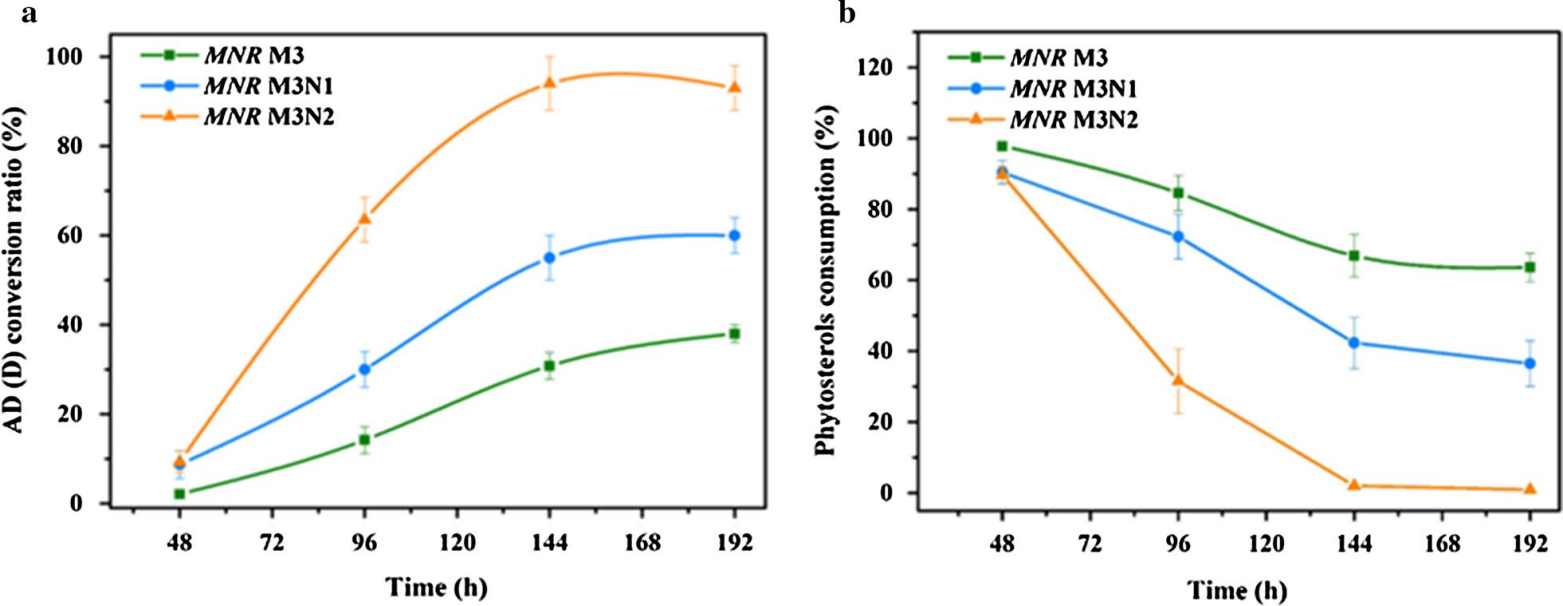
Time courses of phytosterols conversion to AD (D) by *MNR* M3, *MNR* M3N1, and *MNR* M3N2. **a** conversion ratio. **b** Consumption of phytosterols. Tests were conducted with 5 g/L phytosterols, 16% soybean oil and 0.5% tween 80 contained in minimal medium. The *error bars* represent mean ± SD (n = 3)

The recombinant strains both could produce NOX and disturb the redox balance of cofactor (Table 3, Fig. 3), the NAD^+^-dependent pathways were expected to be enhanced in the recombinant strains. To characterize the phytosterols metabolic process of *MNR* M3 in response to the introduction of the NOX: NAD^+^ regeneration system, conversion ratio of major metabolite of AD (D) in *MNR* M3, *MNR* M3N1, *MNR* M3N2 were determined. The bioavailability of steroid hormone in the bioconversion process is low because of the remarkably high hydrophobic nature of steroids. Furthermore, most of the enzymes involved in phytosterols degradation are promoted by oxygen. To improve the production of steroid products in aqueous systems, natural oils have been widely introduced to microbial transformations of steroids [35], as it could increase substrate solubility and improve the dissolved oxygen in steroid biotransformation [21, 36]. So soybean oil was employed here to evaluate the productivity of AD (D) in the constructed strains. As shown in Fig. 4, the time courses of conversion ratio and phytosterols consumption of *MNR* M3, *MNR* M3N1, and *MNR* M3N2 were studied. The highly expressed NOX-2 in *MNR* M3N2 increased the conversion ratio (94%) when compared with that in the parent strain (38%), and the strain with moderate expression of NOX-1 (*MNR* M3N1) increased the conversion ratio (60%) as well. When compared with *MNR* M3, the AD (D) conversion ratio in *MNR* M3N1 and *MNR* M3N2 increased by 58 and 147%, respectively. Moreover, the productivity of AD (D) were analyzed in the phytosterols conversion to AD (D). Among these three strains, *MNR* M3N2 with the highest NOX production showed the highest productivity at every fermentation stage. Moreover, all the substrate of phytosterols (5 g/L) was run out in *MNR* M3N2 fermentation at 144 h. This indicated that the fluxes towards oxidative and reductive metabolism of phytosterols increased upon introduction of NAD^+^ regeneration system in *MNR* M3. Hence, the increase of the NAD^+^/NADH ratio induced by the expression of NOX is important for AD (D) production.

## Discussion

AD and ADD are important steroid medicine intermediates. They are generally obtained via the side chain degradation of phytosterols by microorganisms. The analysis of metabolic pathways associated with AD production from phytosterols showed that either cholesterol oxidase or 3β-hydroxysteroid dehydrogenase is responsible for the first step of the cholesterol degradative pathway, which uses NAD^+^ as a cofactor and oxidizes 3β-hydroxysterols to 3-ketosteroids [37]. Further cleavage of the alkyl sterol side chain at C17 resulted from the fatty acid β-oxidation [20], and the oxidized cofactor is vital for the pathway. Based on the theoretical stoichiometry in AD (D) biosynthesis pathway, one molecule of β-sitosterol is consumed, and 21 molecules of NADH are generated [20, 21]. The high NAD^+^ to NADH ratio could be a promoting factor for the AD (D) conversion ratio from phytosterols through the side chain cleavage. As shown in Fig. 1, both the NAD^+^/NADH ratio and the AD (D) production were increased with the increasing of NA concentration (0–9 g/L). The result was similar to that obtained in the *Torulopsis glabrata* [7], in which the intracellular NAD^+^ level and NAD^+^/NADH ratio increased in the presence of NA.

Cofactor engineering has been widely used to promote the production of important primary metabolites, such as 1, 2-propanediol [10], acetoin [5, 6, 11], and pyruvate [7]. However, the phytosterols degradation process is different from the metabolism of primary metabolites, and is carried out using multi-catabolic enzymes that promote multiple consecutive reactions. Furthermore, the degradation process is closely related to the glucose and fatty acid related metabolic pathways [37, 38]. The complexity of the phytosterols degradation process leads to the limited use of cofactor engineering in AD (D) production. Therefore, powerful technological approaches based on proteomics were used in this study. NADH: flavin oxidoreductase in *MNR* M3 was identified and supposed to the key enzyme to regulate the NAD^+^/NADH balance for AD (D) production through the analysis of comparative proteomic of *MNR* cultured with and without phytosterols (Table 2). The overexpression of NOXs (NOX-1 and NOX-2) in *MNR* M3 was a useful tool for strengthening the phytosterols metabolism and for studying the interaction between the NAD^+^ level and metabolic fluxes. As shown in Figs. 3 and 4, the higher activity of NADH oxidase, the higher NAD^+^/NADH ratio and AD (D) production were got in *MNR* M3N2.

**Fig. 5:**
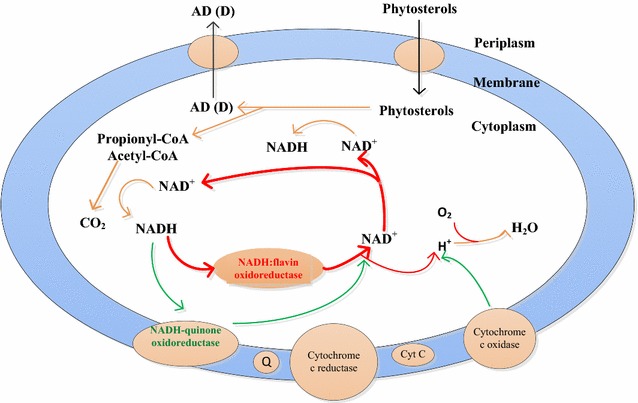
Biotransformation pathways from phytosterols to AD (D) by *MNR* [20, 36, 37]. According to the protein analysis of *MNR*, the red line represents the up-regulated pathway of *MNR* cultured with phytosterols; the green line represents the down-regulated pathway of *MNR* cultured with phytosterols

The large increase of the NAD^+^/NADH ratio due to the introduction of the NAD^+^ regeneration system triggered a dramatic variation of phytosterols metabolism. As shown in Fig. 5, the process from phytosterols to AD that takes NAD^+^, with NADH, propionyl-CoA, and acetyl-CoA, is generated [37, 38]. Moreover, propionyl-CoA and acetyl-CoA are degraded mainly by the tricarboxylic acid (TCA) pathway. So, a sufficient supply of NAD^+^ was essential to the phytosterols degradation pathway [20, 21]. By means of the proteomic analysis, the ETC pathway and NOX-1 with oxygen as the electron acceptor was found important for the regeneration of NAD^+^ from NADH in *MNR* (Table 2). However, NADH-quinone oxidoreductase which was the key enzyme included in ETC pathway, was inhibited, and this finding was consistent with the results of a previous study in which sterol substrates and products (such as ADD and AD) impair the cell’s respiratory chain [29, 30]. Thus, the NOX played an important role in NAD^+^ regeneration for AD (D) production. Finally, the enhancement of intracellular NAD^+^ regeneration system resulted in the strengthening of phytosterols degradation and improvement of AD (D) production and productivity in aerobic fermentation.

## Conclusions

Increasing the intracellular NAD^+^/NADH ratio could be beneficial to the production of AD (D) and that the introduction of NAD^+^ regeneration system into *MNR* is a powerful engineering tool to enhance the metabolic flux for the desired metabolites. The successful expression of NOXs in *MNR* M3 resulted in a large increase in NAD^+^/NADH ratio, thereby markedly enhancing the phytosterols metabolism pathways. The obtained data proved that, besides the manipulation of key enzyme activities, which are included in phytosterols degradation pathways, the maintenance of redox balance and the regeneration activity of cofactors also played an important role in promotion the steroid biotransformation. This study provides a useful strategy for enhancing the accumulation of NAD^+^-dependent microbial metabolites in the microbial biotransformation of steroids.

## Abbreviations

NAD (H): nicotinamide adenine dinucleotides
AD: androst-4-ene-3, 17-dione
ADD: androst-1, 4-diene-3, 17-dione
NOX-1: NADH: flavin oxidoreductase
NOX-2: NADH oxidase
*MNR* M3: *Mycobacterium neoaurum* TCCC 11978
NA: nicotinic acid
DCW: dry cell weight
*kan*: Kanamycin
TMT: tandem mass tag
FDR: false discovery rate
GO: gene ontology
KEGG: Kyoto encyclopedia of genes and genomes
ETC: electron transfer chain
TCA: tricarboxylic acid cycle
